# MEASUREMENT EQUIVALENCE OF THE KESSLER 6 PSYCHOLOGICAL DISTRESS SCALE FOR OLDER ASIAN IMMIGRANT SUBGROUPS

**DOI:** 10.1093/geroni/igz038.2617

**Published:** 2019-11-08

**Authors:** En-Jung Shon

**Affiliations:** Miami University, Oxford, Ohio, Oxford, Ohio, United States

## Abstract

The Kessler 6 (K6) Psychological Distress Scale is a well-known screening instrument to screen for psychological distress of general population. While some studies (e.g., Mitchell & Beals, 2011) concluded that the K6 was appropriate for capturing psychological distress of diverse racial/ethnic groups, other studies (e.g., Andersen et al., 2011) reported that it was less successful in screening for psychological distress of diverse racial/ethnic groups. Few studies conducted measurement equivalence test across older Asian immigrant subgroups. Using Multiple Group Analysis, this study examined whether parameters of the single factor model (items: nervous, hopeless, restless or fidgety, so depressed, everything was an effort, and worthless) is equivalent across the two Asian immigrants (≥65 years; Chinese [n=175] and Korean [n=300] immigrants). Data were generated from the California Health Interview Survey. The configural model showed good fit (X2=41.70 [df=16, p<.001], X 2/df=2.61, CFI=.98, GFI=.97, RMSEA=.06 [90% CI=.04-.08], and SRMR=.04). When all factor loadings were constrained, it indicated measurement non-invariance status between Chinese and Koran (ΔX 2=17.86, Δdf=5, p=.003, CFI=.972, ΔCFI=.009). Given findings of non-invariance on the full constrained model, the invariance test of each factor loading was performed additionally. It was focused on evaluating which items were similar or different across the two groups. The three items, ‘hopeless,’ ‘restless,’ and ‘depress,’ were significantly nonequivalent between the two groups. Clinicians/researchers should aware of the potential risk for misclassification when they try to screen for psychological distress in older Chinese or Korean immigrants. Professionals should pay attention to cross-cultural comparability when interpreting results from the K6.

